# A systematic model identification method for chemical transformation pathways – the case of heroin biomarkers in wastewater

**DOI:** 10.1038/s41598-017-09313-y

**Published:** 2017-08-24

**Authors:** Pedram Ramin, Borja Valverde-Pérez, Fabio Polesel, Luca Locatelli, Benedek Gy. Plósz

**Affiliations:** 1 Department of Environmental Engineering, Technical University of Denmark, Bygningstorvet 115, DK 2800 Kgs, Lyngby, Denmark; 2 Process and Systems Engineering Center (PROSYS), Department of Chemical and Biochemical Engineering, Technical University of Denmark, Building 229, DK-2800 Kgs Lyngby, Denmark; 3 Department of Chemical Engineering, University of Bath, Claverton Down, Bath, BA2 7AY UK

## Abstract

This study presents a novel statistical approach for identifying sequenced chemical transformation pathways in combination with reaction kinetics models. The proposed method relies on sound uncertainty propagation by considering parameter ranges and associated probability distribution obtained at any given transformation pathway levels as priors for parameter estimation at any subsequent transformation levels. The method was applied to calibrate a model predicting the transformation in untreated wastewater of six biomarkers, excreted following human metabolism of heroin and codeine. The method developed was compared to parameter estimation methods commonly encountered in literature (i.e., estimation of all parameters at the same time and parameter estimation with fix values for upstream parameters) by assessing the model prediction accuracy, parameter identifiability and uncertainty analysis. Results obtained suggest that the method developed has the potential to outperform conventional approaches in terms of prediction accuracy, transformation pathway identification and parameter identifiability. This method can be used in conjunction with optimal experimental designs to effectively identify model structures and parameters. This method can also offer a platform to promote a closer interaction between analytical chemists and modellers to identify models for biochemical transformation pathways, being a prominent example for the emerging field of wastewater-based epidemiology.

## Introduction

Models are mathematical representations of real systems that are able to predict their performance under defined conditions. Many chemical and biological systems can be described with stoichiometry and kinetic models. These include differential equations that describe the dynamics of chemical mass or concentrations according to transformation or transfer rates for substrates and products. To predict behaviour of a real system with as small deviation as possible, model structure selection and model parameters estimation should be performed in a systematic procedure referred to as model identification^1^.

Model parameters can be estimated through formal mathematical optimization problems by defining suitable decision variables, namely parameter estimates, objective functions and constraints on, e.g., model parameter values. For the calibration of biochemical pathway models, different types of optimization problems have been defined in systems biology^2^, such as metabolic flux balance analysis for bioprocess optimization^3^ or optimization for biochemical pathways and estimation of kinetic parameters^4^. Moreover, the optimization output (e.g., parameter estimates) could be subject to a significant variability as a result of optimization settings such as parameters uncertainty range. For instance, local optimization methods, such as the flexible Simplex method^5^, can lead to different parameter values depending on the initial guess given as input^6^.

Parameter identification approaches are used to assess uniqueness of a model parameters set, which would result in high prediction accuracy. Parameter identifiability for biological models has been widely studied^7–9^. Typical drawbacks are over-parametrization of models and poor data availability and quality. To overcome parameter identifiability issues the use of effective experimental design has been proposed such as increasing the number of monitored chemicals to improve the parameters identifiability^10^. Additionally, the tasks requires complex parameter estimation protocols, combining several steps, such as sensitivity analysis, parameter correlation, or iterative procedures^7, 11, 12^.

Most water quality models suffer from over-parametrization^13^. Therefore, more recently, a wealth of studies propose robust parameter estimation methodologies and uncertainty analysis in the areas of urban drainage^14, 15^ and wastewater treatment^7, 11, 12, 16^. Techniques proposed for uncertainty and identifiability assessment of model parameters include (i) methods based on local analysis (e.g., Brun *et al*.^7^), which have been demonstrated to be sensitive to the initial parameter value choice^17, 18^; and (ii) global methods, including Monte Carlo-based (MC) methods such as Generalized Likelihood Uncertainty Estimation (GLUE)^19^, or Markov chain- Monte Carlo (MCMC)^20, 21^ based methods. Following model calibration, it is suggested to assess the impact of parameter uncertainty on model outputs. This task can be carried out via MC simulations^22, 23^. However, the propagation of uncertainty between model parameters is not considered in any calibration protocols^12, 24, 25^.

Nevertheless, standardized model identification and calibration procedures are not reported for all biological systems or water quality models. For example, despite the increasing popularity of trace organic micropollutant fate models and their applications in wastewater treatment plants^26–29^ and urban drainage models^29–32^, model parameter uncertainty and parameter identifiability analysis is less well understood. Meanwhile, increased knowledge of transformation pathways has been achieved as a result of extensive experimental efforts^33, 34^. These challenges are particularly relevant when the combined identification of transformation pathways and estimation of transformation rates are required. More specifically, in the emerging field of wastewater-based epidemiology (WBE) – in which concentrations of drug biomarkers measured at sewer outlets are used to back-calculate drug consumption in urban areas – fate models have been only recently introduced^31, 35–37^ to account for in-sewer transformation of drug biomarkers. In these approaches, back-calculation of drug consumption is directly linked to the in-sewer transformation rates of drug biomarkers during transport. Biased prediction of transformation rates or associated uncertainties can result in inaccurate drug consumption estimates.

Estimation of reliable uncertainties is even a bigger challenge once more complex fate models are used, such as those combining primary (e.g., fate of organic carbon fractions or microbial growth) and secondary metabolic processes (transformation of trace organic chemicals, e.g., drug biomarkers or pharmaceuticals)^38–40^. A knowledge gap exists in identifying metabolic transformation pathways in complex biological matrices – a focal area chosen for the present study. For instance, cocaine in wastewater is transformed to benzoylecgonine and other metabolites, different from those undergoing through human metabolism. The task is even more challenging, for newly synthetized drugs, for which limited or no information about their human and in-sewer bacterial metabolism exists. The identification of major drug transformation pathways is necessary to close mass-balances in multi-level transformation routes. We note that measuring drug biomarkers, at the ng L^−1^ to µg L^−1^ range, is a labour-intensive and costly task, which involves quantitative analysis of target compounds that have to be selected prior to experiments. The selection should be performed through proper and efficient assessments.

The main goal of this study was to develop a model identification method for biochemical transformation pathways by employing sound parameter uncertainty propagation. The method was benchmarked against selected conventional techniques in terms of prediction accuracy, transformation pathway identification efficiency and parameter correlation. The method is applied to identify transformation pathways for the human biomarkers of the illicit drug heroin in wastew﻿ater, accounting for both biotic and abiotic transformations.

## Results and Discussion

### Model identification methodology

**Figure 1:**
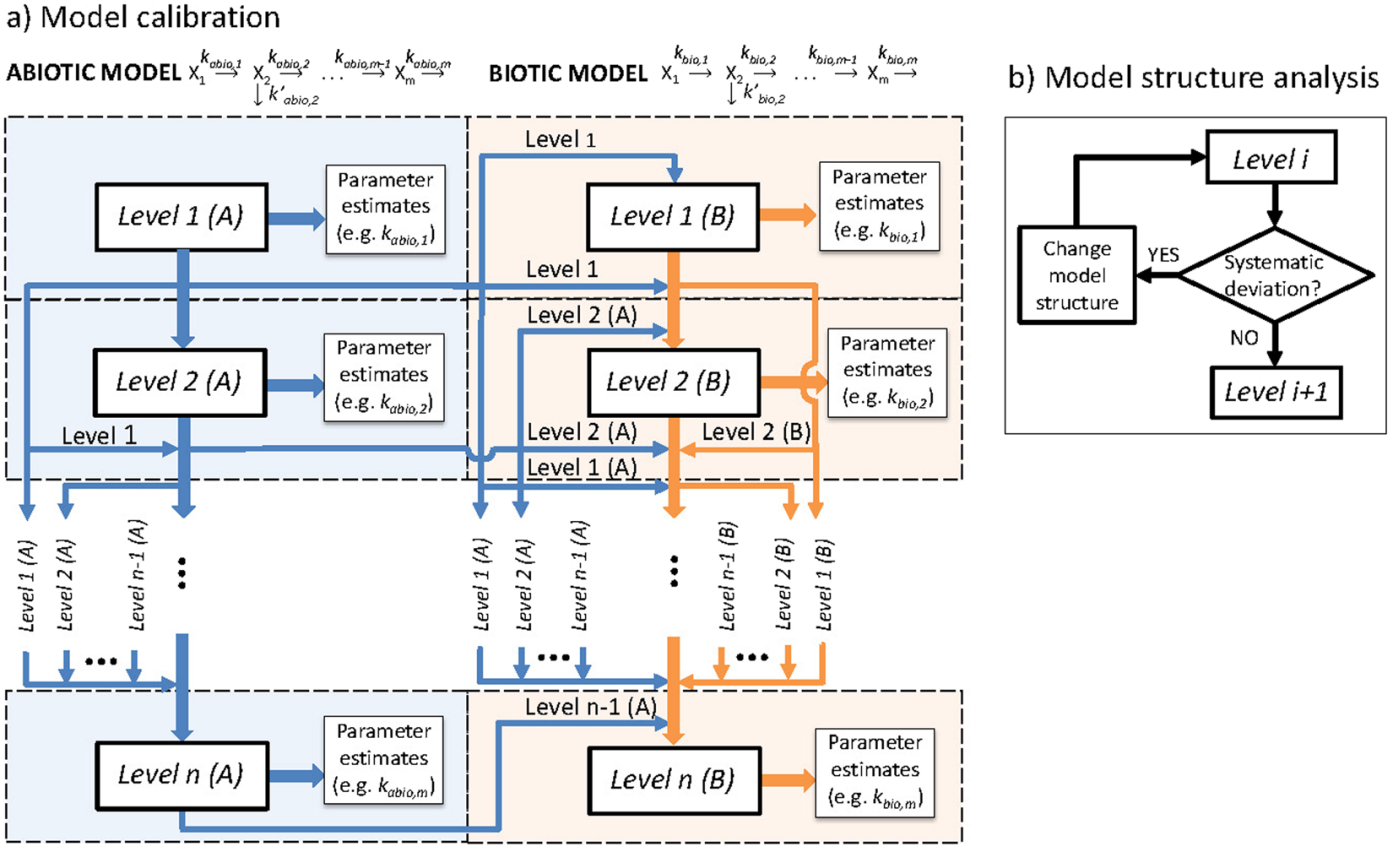
(**a**) Overview of the proposed identification method, referred to as *Method 1* in this study, to estimate transformation rate constants in metabolic pathway models for both abiotic and biotic processes. The method includes *n* calibration levels for *m* number of metabolites (i.e., *X*_*1*_ to *X*_*m*_) and *m* + *1* number of parameters for abiotic model (i.e., *k*_*abio*,*1*_ to *k*_*abio*,*m*_ and *k*′_abio,2_) and biotic model (i.e., *k*_*bio*,*1*_ to *k*_*bio*,*m*_ and *k*′_bio,2_). The method starts from *Level 1* (abiotic) and ends at *Level n* (biotic). Each level includes 2–3 calibration steps. Arrows indicate the information flow, i.e., parameter uncertainty propagation, among the levels. Blue and orange arrows pass the information from abiotic (*A*) and biotic (*B*) model parameters, respectively. (**b**) Additional evaluation step between any two levels to assess the accuracy of model structure by detecting systematic deviations from experimental results.

An overview of identification methodology is presented in Fig. 1a. The method involves three stages: (I) estimation of parameters at defined levels; (II) evaluation of model structure (i.e., kinetic and/or pathway) at each level; and (III) propagation of parameters uncertainty to the subsequent levels. The estimation methodology consists of *n* levels, defined according to assumed abiotic (A) and biotic (B) reaction pathways. The chemical pathways are represented by *m* chemicals (from *X*_1_ to *X*_*m*_) transformed via abiotic (e.g., hydrolysis) and microbially-mediated reactions. Kinetic model parameters thus include *m* + *1* abiotic transformation rates, represented by *k*_*abio*,*1*_ to *k*_*abio*,*m*_ (d^−1^)﻿ and *k*′_abio,2_ (d^−1^), and *m* + *1* biotic transformation rate constants, represented by *k*_*bio*,*1*_ to *k*_*bio*,*m*_ (L gTSS^−1^ d^−1^)﻿ and *k*′_bio,2_ (L gTSS^−1^ d^−1^). To simplify the representation, the number of transformation rates for abiotic and biotic processes are considered the same.

Model parameters at each level are considered to be either *primary*, *subsidiary* or *combinatorial*. *Primary* parameters are the ones introduced at any level for the first time by employing priors in the form of even parameter probability distributions set with arbitrary or e.g., literature-informed ranges. *Subsidiary* parameters are the ones previously estimated at upstream levels and which are relevant to parameter estimation at downstream levels in terms of uncertainty propagation. *Combinatorial* parameters are similar to *primary* ones, and are associated with chemicals, exhibiting two transformation pathways. In other words, *combinatorial* parameters define the overall transformation rate of the chemical and are equal to the sum of transformation rates to each product. Each of the transformation rates are turned into *primary* parameters in the following level. For instance, from the example shown in Fig. 1, at level 1(A) *k*_*abio*,*1*_ (arbitrary boundaries, uniform distribution) is a *primary* parameter. At level 2 (A) previously estimated *k*_*abio*,*1*_ (known boundaries and distribution inferred at level 1) is a *subsidiary* parameter and “*k*_*abio*,*2*_ + *k*′_*abio*,*2*_” (arbitrary boundaries, uniform distribution) is a *combinatorial* parameter. At level 3 (A), *k*_*abio*,*1*_ (boundaries and distribution inferred at level 1) remains as *subsidiary* parameter whilst *k*_*abio*,*2*_ (known upper boundary inferred from previous *combinatorial* parameter estimation, uniform distribution), *k*′_*abio*,*2*_ (known upper boundary inferred similar to *k*_*abio*,*2*_, uniform distribution) and *k*_*abio*,3_ (arbitrary boundaries, uniform distribution) are all *primary* parameters, in case there is no extra branch for *X*_3_.

Each level consists of a three-step parameter estimation approach: (i) *Prior settings*: prior model parameter distribution ranges are defined by either considering uniform distribution for *primary* parameters or a distribution function for *subsidiary* parameters, as indicated in Fig. 1; (ii) *Global optimization*: global optimum parameter values are estimated using an iterative/adoptive algorithm, e.g., DREAM_(ZS)_^41^ in present study; (iii) *Post processing*: The range of uncertainty and type of distribution of posterior *primary* parameters are determined, Supplementary Table S3. Once a unique set of parameter is estimated, deviations of model predictions from measurements are assessed. In the case of systematic error in model prediction, the structure of kinetic model (e.g., second-order or of first-order kinetics) and the chemical transformation pathways (e.g., new transformation products) are re-evaluated (Fig. 1b). This iterative approach ensures that uncertainty of parameters would only propagate when the model structure is adequately described.

In Fig. 1, the arrows represent the information flow. Whilst the distribution from abiotic to biotic model is considered for all calibration levels (blue arrows), the biotic calibration levels only distribute the information (orange arrows) within the calibration levels for biotic processes (e.g., the information does not pass from *Level 1*(*B*) to *Level* 2(*A*)). Although Fig. 1 presents the methodology for a simple chain pathway, the method can be applied for multi-branched pathways including parallel or consecutive transformations.

### Case study of heroin and codeine transformation pathways

**Figure 2:**
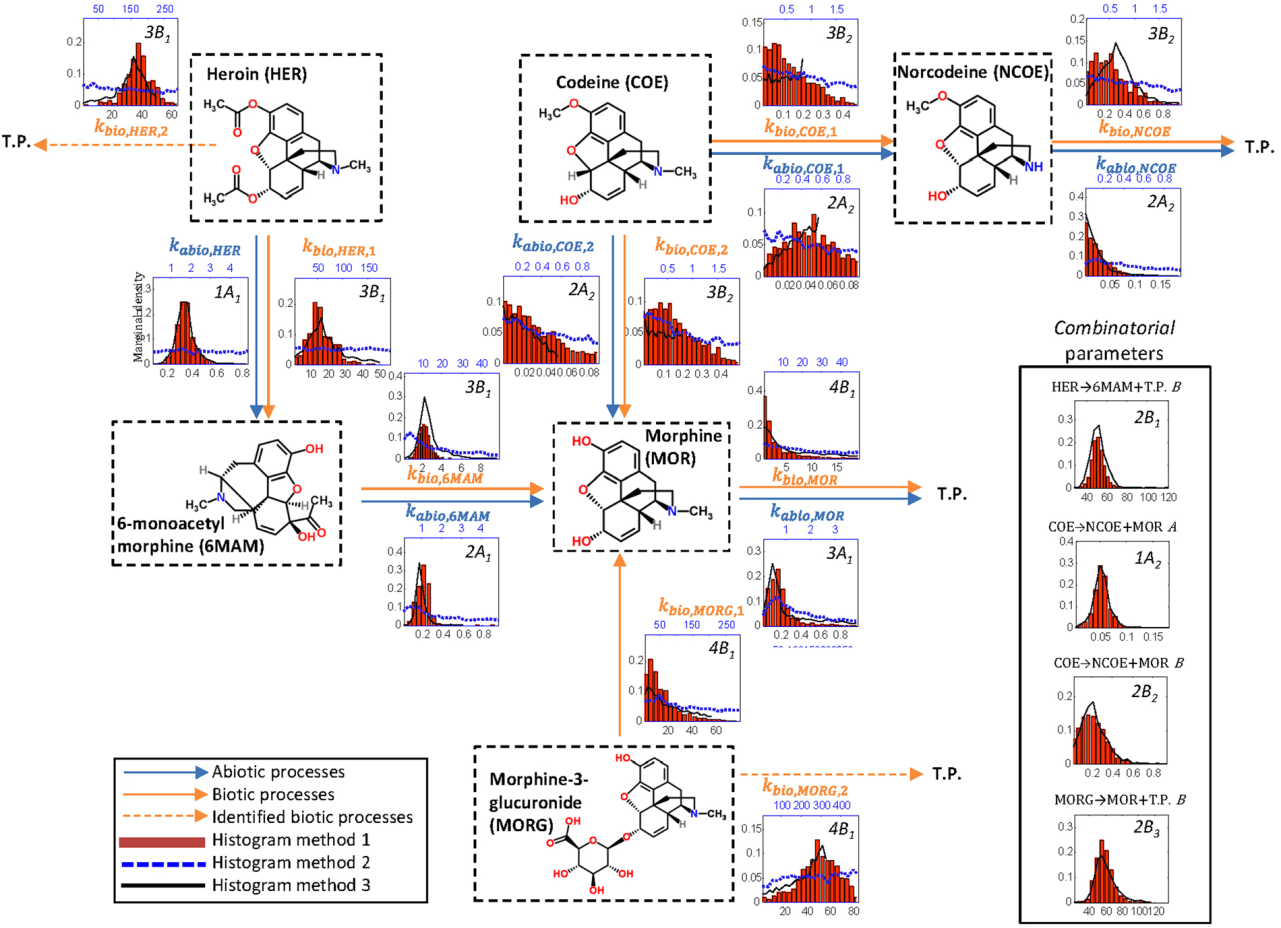
The identified transformation pathway of heroin (HER) and codeine (COE) drug biomarkers, including abiotic and biotic processes. Posterior distribution of estimated parameters (histograms) for proposed methodology – *Method 1* (in red), *Method 2* (dotted blue line, upper X axis) and *Method 3* (solid black line) at 4 different calibration levels. For *Method 2* and *3*, distributions are shown with linear interpolations between pairs of distribution data points. Abbreviations: T.P. - unknown transformation product (TP); A - Abiotic model, B - Biotic model. Stoichiometry and process rates are according to transformation pathway of HER and COE drug biomarkers and are shown in the Supplementary Table S1. Solid arrows are pathways identified from literature and new identified pathways are shown with dashed arrows.

The transformation pathway for heroin (HER) and its human metabolites 6-monoacetylmorphine (6-MAM), morphine (MOR), and morphine-3-β-D-glucuronide (MORG), and codeine (COE) and its human metabolite norcodeine (NCOE) are depicted in Fig. 2. The pathway model was derived based on former studies^38, 42^ employing pathways primarily based on human metabolism, including the transformation of COE to MOR. Following the kinetic model calibration, the overall transformation pathway was extended with two additional pathways/branches for HER and MORG (dashed arrows). A kinetic model based on Activated Sludge Modelling framework for Xenobiotics (ASM-X)^31^ was used to describe the drug transformations (Supplementary Table S1). In the present study, the assumption of accurate kinetic model to predict removal or formation of each individual drug biomarkers, has simplified the task of identification of model structure to only pathway identification.

New pathways were initially hypothesized by checking the mass balances in the pathway at any given time (i.e., the amount of transformation of a compound to another should correspond to formation of the second compound). To apply the model identification methodology, the concentration profiles of these chemicals from a targeted batch experiment in wastewater were used^38^.

### Estimation of transformation rates for the case study

Applying the novel parameter estimation method to the case of heroin and its biomarkers resulted in a four-level model identification problem, including seven abiotic model parameters (*A*) and nine biotic model parameters (*B*) (Fig. 2). As compared to the pathway elucidated in Fig. 1, this pathway has an unequal number of *k*_*abio*_ and *k*_*bio*_ parameters (as a result of pathways adopted from human metabolism and also two new additional pathways). The posterior distributions (histograms) of estimated *primary* parameters (Fig. 2; Supplementary Fig. S1) are presented along with the transformation pathways, thereby indicating the information flow direction. Four additional histograms for *combinatorial* parameters are also shown separately for COE (abiotic and biotic), HER (biotic) and MORG (biotic) (Fig. 2). Model calibration was initiated by the estimation of *k*_*abio*_ for chemicals not formed from other chemicals – HER and COE (Fig. 2, denoted by *1*(*A*)). Below, each identification level is briefly described, with ranges and distributions of all parameters reported in Supplementary Table S3.

*Level 1*(*A*). To estimate *k*_*abio*,*HER*_, a uniform probability distribution and an arbitrary range were considered as prior information. Measured concentrations of MORG during abiotic experiments showed no transformation, hence *k*_*abio*,*MORG*_ was set to zero. In parallel, *combinatorial* parameter *k*_*abio*,*COE*_ (*k*_*abio*,*COE*,*1*_ + *k*_*abio*,*COE*,*2*_) was estimated.

*Level 2*(*A*) *and 2*(*B*). This level includes the estimation of the *primary* abiotic parameter for 6MAM (*k*_*abio*,*6MAM*_), involving the *subsidiary* parameter, *k*_*abio*,*HER*_. Additionally, for COE and NCOE, the estimation involves (*k*_*abio*,*COE*,*1*_, *k*_*abio*,*COE*,*2*_ and *k*_*abio*,*NCOE*_) that includes the *subsidiary* parameter *k*_*abio*,*COE*_. Biotic *combinatorial* parameters for overall MORG biotransformation, *k*_*bio*,*MORG*_ (*k*_*bio*,*MORG*,*1*_ + *k*_*bio*,*MORG*,*2*_), overall HER biotransformation, *k*_*bio*,*HER*_ (*k*_*bio*,*HER*,*1*_ + *k*_*bio*,*HER*,*2*_), and overall COE biotransformation, *k*_*bio*,*COE*_ (*k*_*bio*,*COE*,*1*_ + *k*_*bio*,*COE*,*2*_), were also estimated at this level.

*Level* 3(*A*) *and 3*(*B*). Abiotic *primary* parameter, *k*_*abio*,*MOR*_, and the rest of abiotic model parameters were considered *subsidiary*. Biotic *primary* parameters were also estimated (i.e., *k*_*bio*,*HER*,1_, *k*_*bio*,*HER*,*2*_ and *k*_*bio*,*6MAM*_). Additionally, the biotic *primary* parameters *k*_*bio*,*COE*,*1*_, *k*_*bio*,*COE*,*2*_ and *k*_*bio*,*NCOE*_ were estimated. Relevant *subsidiary* parameters from previous levels were considered at each estimation.

*Level 4*(*B*). The remaining three biotic model parameters, i.e., *k*_*bio*,*MORG*,1_, *k*_*bio*,*MORG*,2_, and *k*_*bio*,*MOR*_, were estimated using all the other model parameters as *subsidiary*.

### Comparison with conventional model parameter estimation methods

Two parameter estimation methods, referred to as *Method 2* and *Method 3*, were used to benchmark *Method 1*, and to assess the impact of different uncertainty propagation approaches on parameter estimates.

*Method 2*: The method, also referred to as the lumped approach, comprises the estimation of all model parameters using a single objective function, in which all seven *k*_*abio*_ were estimated simultaneously using only the abiotic experimental data and all nine *k*_*bio*_ were estimated simultaneously using only the biotic experimental data. Examples of the application of *Method 2*, whereby a parameter subset is calibrated to fit several experimental data series, are common in literature^6, 12, 16, 24, 43^. To apply this method in this case study, we propagated uncertainties from the abiotic model to biotic ones (i.e., by employing abiotic parameters as *subsidiary*). Therefore we considered the 95% credibility interval of the abiotic estimated parameters as uncertainty range for the estimation of all nine *k*_*bio*_ corresponding to the biotic model.

*Method 3*: The method consisted of the estimation of parameters in a sequential order, similar to *Method 1*. However, the propagation of parameter uncertainties was omitted, i.e., at each level, fixed values of the already estimated parameters were used as priors (see, e.g., Insel *et al*.^44^). Thus, all parameters in *Method 3* are treated as *primary*. This is a common approach for the calibration of, e.g., nitrous oxide (N_2_O) production models, in which parameters only related to N_2_O are estimated and the remaining parameters are fixed^45^. However, it has been demonstrated that by ignoring the uncertainties introduced by fixing parameter values may lead to significant underestimation of the uncertainty in emissions of N_2_O^46^.

**Figure 3:**
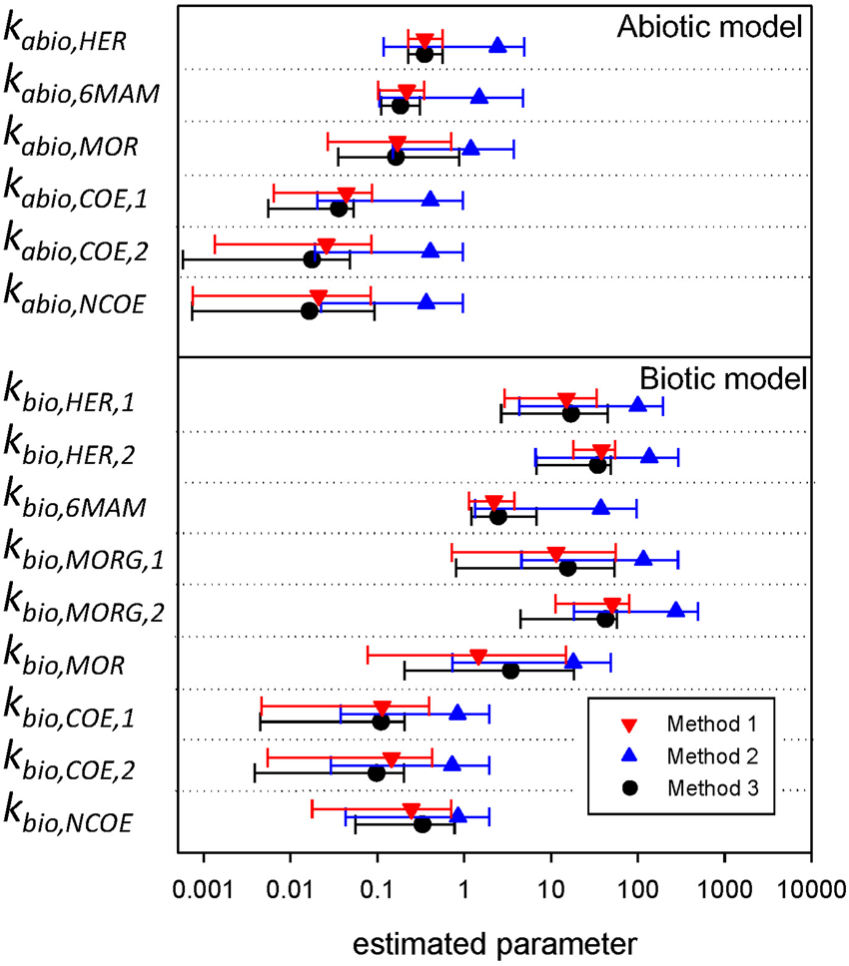
Values of estimated parameters — *k*_*abio*_ (d^−1^) in abiotic model and *k*_*bio*_ (L gTSS^−1^ d^−1^) in biotic model—using *Method 1 (proposed methodology)*, *Method 2* (lumped approach) and *Method 3* (no propagation of uncertainties). Marks are estimated values (median) and error bars represent 95% credibility interval (lower bound, upper bound). Abbreviation: T.P. = transformation product(s).

The histograms from *Method 1* and *Method 3* (Fig. 1, red bars and solid black lines) show that for most parameters the posterior distribution are skewed (e.g., significant positive skewness for MOR (B_1_)). A comparison of the estimated parameter values (median) and uncertainty of parameter estimates reported as 95% credibility interval is presented in Fig. 3. These results indicate comparable range and similar distribution for parameter sets obtained with *Method 1* and *Method 3*. This is not the case for most of the parameters subsets obtained using *Method 2*, which leads to comparably higher parameter values and predominantly wider uniform distributions. We note, however, that the wider parameter ranges are a result of the wider boundaries considered for *Method 2* (Fig. 2), thereby ensuring no restriction of solution space that could potentially include a global optimum. Using *Method 2*, the uniform posterior distribution shows poor parameters identifiability as different combination of parameter values could result in similar prediction accuracy.

The discrepancy between ranges of parameter estimates obtained using *Method 1* and *Method 3* is at maximum for *k*_*bio*,*6MAM*_ in which range of this parameter from *Method 3* is more than twice of the width of the range estimated by *Method 1*. However, the range of *k*_*bio*,*COE*,*2*_ estimated by *Method 1* is almost twice of that estimate by *Method 3*. In addition, for some parameters the posterior distribution from *Method 1* and *Method 3* are different (Fig. 2), especially for the primary parameters that are estimated based on a *combinatorial* parameter e.g., *k*_*abio*,*COE*,*1*_ and *k*_*abio*,*COE*,*2*_ at level 2.

**Figure 4:**
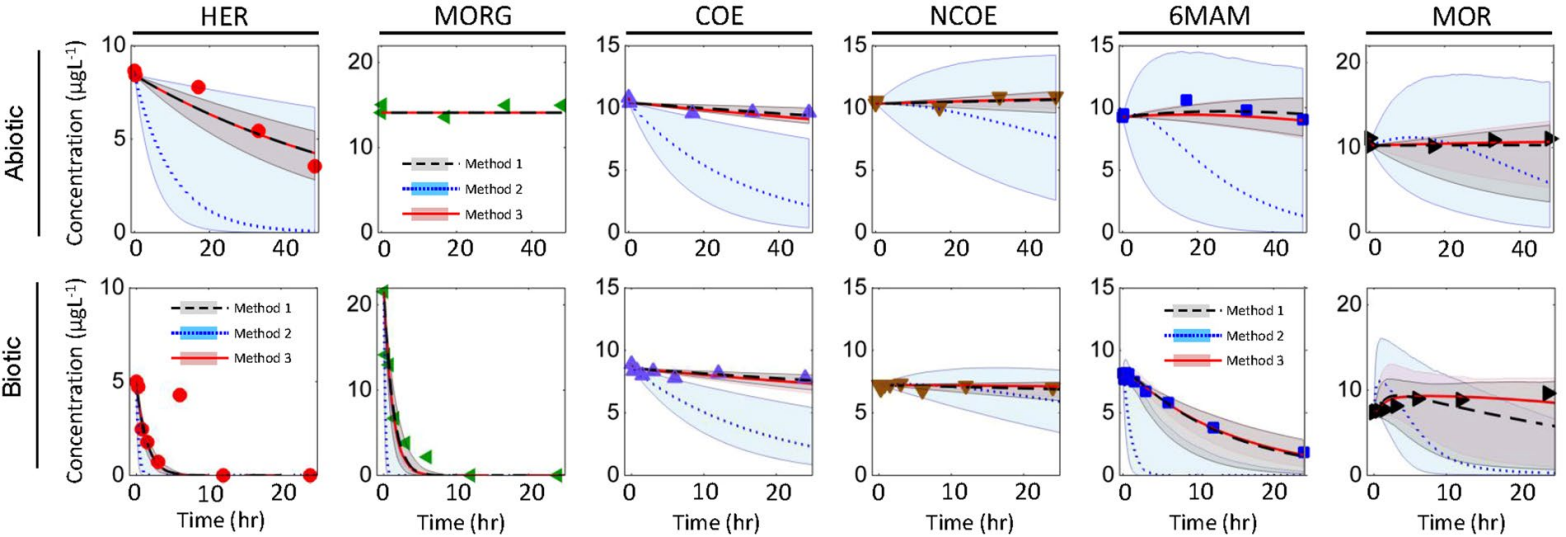
Measured and simulated biomarker concentration data with uncertainty bands obtained using *Method 1*–*3*. Markers are measured data and lines are simulation results. The shaded area reflects 95% credibility interval of model prediction (red area and full line: *Method 1*, grey area and dashed line: *Method 2*, blue area and dotted line: *Method 3*).

Following parameter estimation, the impact of parameter uncertainty on model outputs (i.e., drug concentrations) was assessed through uncertainty analysis using Monte Carlo simulations (Fig. 4). These results suggest that *Method 2* can lead to estimates with higher uncertainties for both abiotic and biotic model parameters. Notably, estimated parameter values and associated uncertainties obtained using *Method 2* are highly dependent on the choice of the parameter ranges used in the optimization, i.e., higher ranges would result in higher estimates calculated as the mean of often uniform posterior distributions.

The accuracy of model predictions derived using the three different calibration methods was assessed using statistical tests (Table 1). Values obtained for the Root Mean Squared Error (RMSE) and the Mean Absolute Error (MAE) are related to model prediction accuracy: the lower the RMSE and MAE are the more accurate the model prediction is. Additionally, the average relative Interval Length to Coverage of measurements by prediction bands (ILTC) was used for accuracy assessment. Lower ILTC is an indication of lower prediction uncertainty together with higher coverage of measured values by uncertainty band. Results obtained using these statistical tests (Table 1) suggest that, for the abiotic and biotic model, *Method 2* was the least accurate for all parameters, reporting, on average, 8 times higher RMSE and MAE values and 5 times higher ILTC values compared to *Method 1* and *Method 3*. We note that comparably high coverage of measurements were obtained using *Method 2*, which does not necessary translate into high accuracy. Results obtained for HER using *Method 1* showed similar parameter estimation accuracy to that obtained with *Method 3*. For abiotic model, higher accuracy was obtained using *Method 1* for NCOE and MOR. Interestingly, *Method 3* showed slightly higher accuracy for COE and 6MAM compared to *Method 1* for the abiotic model. As for the biotic model, the prediction accuracy obtained using *Method 1* and *Method 3* showed comparable performance except for MOR and 6-MAM, for which *Method 1* resulted in higher accuracy, and for MORG, *Method 3* showed a better performance.

**Table 1:**
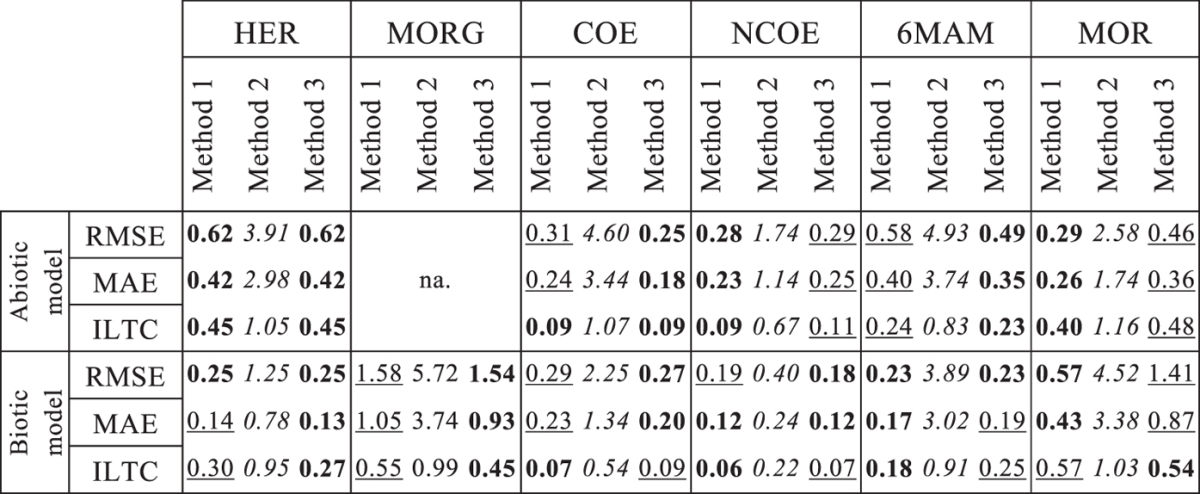
Assessment of simulation accuracy of models calibrated using *Methods 1*–*3*. Abbreviations: RMSE, root mean squared error; MAE, mean absolute error; ILTC, interval length to coverage. For better comparison among the three methods following color code is used: boldface – high accuracy; underlining- moderate accuracy; *italic type* – low accuracy.

To assess the significance of uncertainty propagated via *subsidiary* parameters to primary *parameter* estimates, a global sensitivity analysis (GSA) was performed according to standardized regression coefficients (SRC) method^47^ (Supplementary Fig. S2). These results show the importance of propagated uncertainty at each calibration level. It was found that less uncertainty propagation can be expected from the subsidiary parameters located at the further upstream of a calibration level. Nevertheless, since in present case study all chemicals in the pathway and their formation or transformation kinetics are required for back-calculation in WBE studies, no parameter subset selection was performed based on GSA results, common practice in most calibration protocols^8^.

We note that the choice of SSE as objective function may have had significant impact on the convergence of the optimization algorithm for parameter estimation as we used equal weight factors in all methods. There are methods proposed to assess the impact of weights combinations on optimization resuts^48^. The effect of selection of the objective function on parameter estimation is not further analysed in this study, as it has been previously reported by Hauduc *et al*.^49^.

### Analysis of parameter correlation

**Figure 5:**
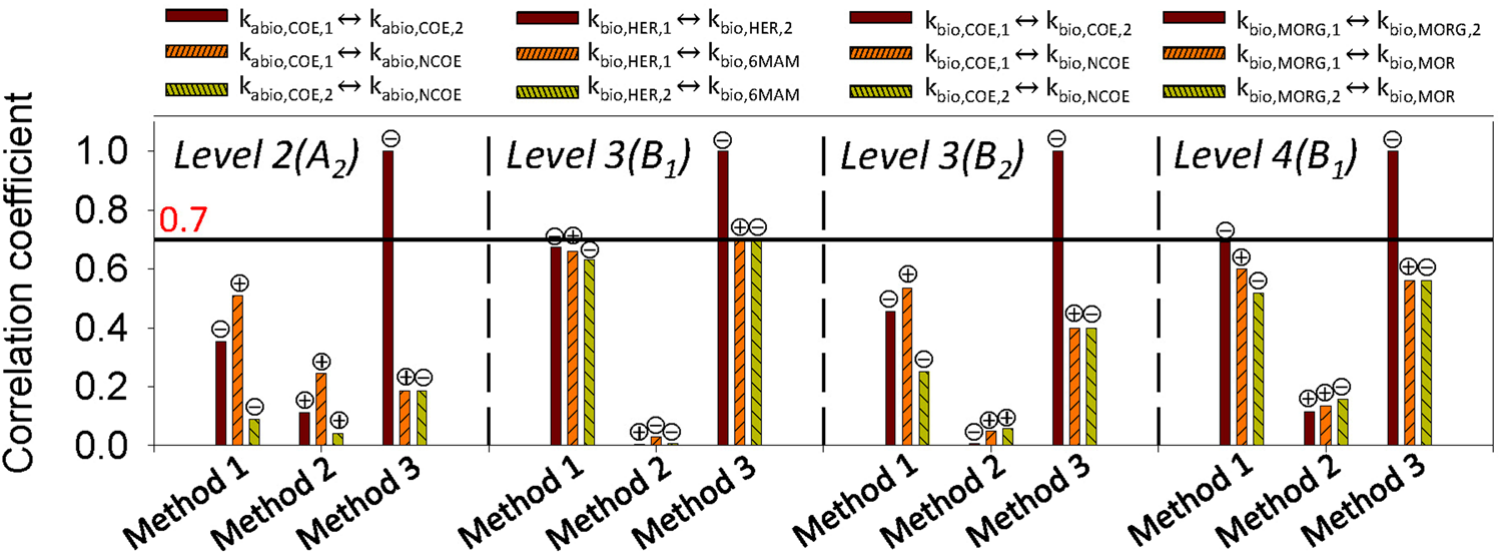
Linear correlation coefficients for model parameters following parameter estimation in calibration *Method 1*–*3*. Correlation threshold for identifiability defined at 0.7 according to Frutiger *et al*.^50^. Positive and negative correlations are designated with + and − respectively.

To assess the impact of the parameter estimation methods on parameter collinearity, correlation analysis was performed on the posterior parameter distributions using *Methods 1*–*3* (Supplementary Figs S3–5﻿). According to the linear correlation coefficients (LCC) obtained and considering a collinearity threshold for identifiability to be 0.7^50^, all estimated parameters from all methods were identifiable except for parameters related to the chemicals with two transformation branches in *Method 3* (i.e., HER and MORG transformation pathways, Fig. 2). Figure 5 shows the collinearity index for those parameters that are highly correlated according to *Method 3* together with the collinearity index from *Method 1* and *2* for further comparison. The sign of correlations indicates whether the parameters are positively or negatively correlated.

Results suggest that *Method 1* typically resulted in increased values of LCC compared to *Method 2*, which results from incorporating uncertainties propagated from other upstream model parameters. Using *Method 3* maintained a good model-fit for the corresponding chemicals (HER abiotic; COE, NCOE, abiotic and biotic) by fixing the sum of kinetic parameters from each branch (a hard optimization constraint, e.g., *k*_*bio*,*HER*,*1*_ = *k*_*bio*,*HER*_ − *k*_*bio*,*HER*,*2*_). However, perfect inverse linear relationship (correlation: −1) was obtained for the transformation rate constants for abiotic HER as well as for abiotic and biotic COE, NCOE transformation, implying that *k*_*bio*,*HER*,*1*_ can be compensated by decreasing *k*_*bio*,*HER*,*2*_. This non-identifiability problem did not appear in the *Method 1* by optimizing kinetic parameter of each transformation at the same time, whilst the optimization is constrained to maintain the sum of the *primary* parameters within the 95% credibility interval of *combinatorial* parameter (a soft optimization constraint). All parameters obtained using *Method 2* showed very weak correlations, as the optimizer could not converge to any particular optimal point.

### Identifying new transformation pathways

**Figure 6:**
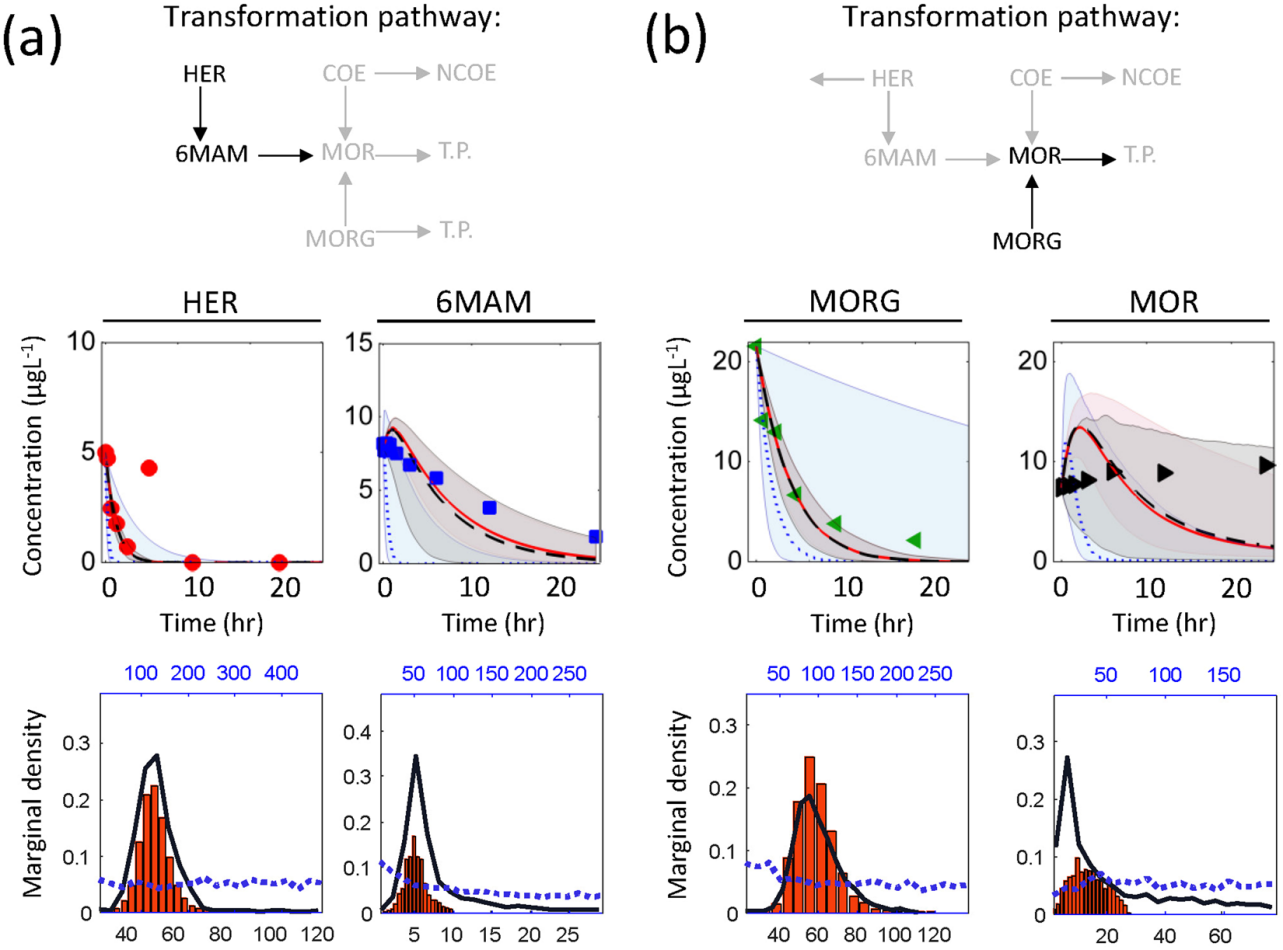
Biomarker transformation pathway identification considering human metabolism as prior knowledge. Pathways for HER and 6MAM (**a**) and MORG, MOR (**b**) are highlighted. Simulation results are demonstrated for highlighted chemicals using calibration *Method 1*–*3*. Posterior parameter probability distributions (i.e., histograms) were obtained using *Method 1* (in red), *Method 2* (dotted blue line, upper X axis) and *Method 3* (solid black line).

The transformation pathway network illustrated in Fig. 2 includes two newly identified pathways for HER and MORG via biocatalysis in wastewater as compared to human metabolism^42, 51^. These pathways were identified using mass balance calculations over the transformation of 6MAM and MOR, and were compared with available rule-based data base^52^. If the biomarker transformation pathways in wastewater would follow human metabolism, i.e., no additional transformation for HER and MORG, a significant discrepancy would be obtained between measured and predicted data (Fig. 6). Only based on measured data, one cannot infer the existence of another pathway with sufficient evidence, since net accumulation of chemicals depends on both transformation and formation rates. Hence, one way to assess whether an additional pathway exist, is to estimate transformation rates before pathway modification and assess the uncertainty of transformation rates on predictions. Figure 6 attempts to assess if the information on additional pathway can be inferred using *Methods 1*–*3*.

If 6MAM is the sole transformation product of HER, rapid transformation of HER should be followed by the formation of 6MAM in the first 3 hours of experiment. However, the measurements for 6MAM do not show accumulation (Fig. 6a). Model predictions for MORG and MOR simulation results following calibration using *Method 1* and *Method 3* showed rapid net formation of MOR during the simulation time (Fig. 6b). However, this trend does not agree with measured data. In contrary, *Method 2* suggests very high removal rate. Figure 6 additionally reports the distribution of posterior parameter values obtained using *Methods 1*–*3*. Histograms indicate that parameters estimated using *Method 1* and *3* are identifiable (clear peak shape), whilst *Method 2* failed to show any particular distribution. Following this assessment, the model structure (i.e., the pathways) was modified to eliminate the systematic deviation imposed by inaccurate pathway model.

By comparing parameter ranges from *Method 1* and *Method 3* (Fig. 3), for the newly identified pathways, it emerges that *Method 3* resulted in 14% higher uncertainty range for transformation rate *k*_*bio*,*HER*,*2*_, whilst 28% higher uncertainty range was estimated for *k*_*bio*,*MORG*,*2*_ using *Method 1*. It is reported that for identifiable parameters, the relative parameter estimation error, such as 95% confidence interval, should be less than 50% of an estimate^50^. Based on this criterion, none of the parameters related to the new pathways were identifiable i.e., lowest confidence interval was 52.5% (for lower bound) for *k*_*bio*,*HER*,*2*_ using *Method 1*.

High uncertainty ranges reported in Fig. 3 correspond to significant skewedness of the histograms. Nevertheless, the two additional pathways found in this study are in agreement with existing literature, in which incomplete deconjugation of glucuronide metabolites to the respective parent substance was shown in wastewater for morphine^53^, lamotrigine^54^ and sulfamethoxazole^55^.

### Outlook

The main objective of this study was to develop an identification method for reaction kinetics and multi-branched chemical transformation pathways models. The proposed approach employs uncertainty propagation between model parameters. Based on the results obtained, the prediction accuracy, transformation pathway identification and parameter identifiability, the benefits of sound uncertainty propagation using *Method 1* seem to be significant compared to the other two methods. The results show that sound uncertainty propagation may lead to even more accurate model prediction.

It is not certain that model parameters can be estimated unambiguously, if the dynamic model is only partially observable (e.g., undetermined concentrations in the pathway) due to technical limitations^56^. Therefore, modifying existing transformation pathway models to find pathway gap is often necessary. Results obtained in this study indicate that the proposed parameter estimation method can be useful to identify transformation pathway branching and the associated reaction kinetics. New transformation pathways proposed based on the method should be now confirmed by analytical chemists by characterizing the transformation product.

As for the practical aspects, to effectively select biomarkers to be used in targeted experiments, identifying transformation pathways of xenobiotics in complex biological matrices, such as wastewater, has a particular relevance for WBE^57^. The present study can serve as an input for optimal experimental design (e.g., stability tests or pathway identification), as currently there is little information available to address this issue. It can be used to assess the inclusion of all the necessary chemicals presented in the pathway, which would facilitate the identification of model parameters. Moreover, estimated transformation rates could be helpful to re-define sampling strategy during batch experiment. For reliable back-calculation of substance (e.g., drugs) consumption rates in urban areas, WBE engineering approaches will be required to employ biokinetics and transport models. In this context, biased kinetic rate uncertainty can lead to unreliable predication uncertainty. Overall, our method can fill such a gap by providing a practical approach to identify potential transformation pathways and transformation kinetics models. Model validation, which is an essential step to verify the accuracy of a calibrated model, is not presented here as part of the model identification procedure. In fact if the model fails to be validated with an independent dataset, the experimental design, model structure analysis and model parameters should be evaluated or estimated again. Nevertheless, model validation results for 6MAM have already been presented in our previous study^38^. It should be noted that, back-calculation for WBE studies is performed at sewer catchment level, and this requires identification of a reactive transport sewer model to reliability estimate chemicals conversions in the sewer during their hydraulic residence time. Hence, inclusion of uncertainties related to sewer predictions should be also accounted for^36^.

## Methods

### Parameter estimation method

In this study, a method is developed to propagate information through transformation pathway levels, including *a priori* parameter probability ranges and distribution. For Level 1, a uniform prior distribution is assumed with an arbitrary parameter range based on preliminary assessments. For subsequent levels, the 95%-credibility interval of the parameter estimates identified at each Level (e.g., *k*_*abio*,*HER*,*1*_ at *Level 1*(*A*_*1*_)) is considered as the uncertainty range for all subsequent Levels (e.g., *Level 2(A)*_1_ and *Level 2*(*B*_1_)). The distributions of posterior parameters were identified by testing different distribution functions including *beta*, *birnbaumsaunders*, *exponential*, *extreme value*, *gamma*, *generalized extreme value*, *generalized pareto*, *inversegaussian*, *logistic*, *loglogistic*, *lognormal*, *nakagami*, *normal*, *rician*, *tlocationscale* and *Weibull* distributions. To identify probability distributions, the closest fit was found using *allfitdist* in Matlab R2014a (Mathworks, US).

### Optimization method

Model parameters were estimated using the Bayesian optimization method Differential Evolution Adaptive Metropolis (DREAM_(ZS)_)^41^, employing the normalized sum of squared error (SSE) as the objective function:

1 $$SSE={\sum _{i=1}^{n}\sum _{j=1}^{m}(\frac{{\hat{y}}_{i,j}-{y}_{i,j}}{{\hat{y}}_{i,j,{\rm{\max }}}-{\hat{y}}_{i,j,{\rm{\min }}}})}^{2}$$

where *n* is the number of measurements series, *m* the number of the data points in each series, $\hat{y}$ is the measured data and *y* the model predictions. ${\hat{y}}_{i,jmax}$ and ${\hat{y}}_{i,jmin}$ indicate maximum and minimum of measurements, respectively. Objective function included all the model outputs (i.e., simulated concentrations) and measured values up to each level. DREAM_(ZS)_ was employed using 5 Markov chains in parallel with 20000–50000 maximum number of function evaluation for each estimation. Following optimization, only the posterior parameters that resulted in good agreement between model output and measured data were selected for further analysis based on Theil inequality coefficient (TIC), with acceptable threshold of 0.3^58^. Besides the DREAM algorithm - used in this study, other optimization approaches can also be used to estimate model parameters such, e.g., Latin Hypercube Sampling based Simplex (LHSS)^6^.

### Benchmarking the model identification method developed

We benchmarked the developed approach (*Method* 1) by comparing it with two methods based on literature studies:

*Method 2*: Parameters are estimated in a concerted way and by omitting *a priori* information regarding the range and the parameter probability distribution – also referred to as the “lumped” estimation method. To estimate the biotic model parameters, *a priori* information related to the abiotic parameter range is considered as 95% credibility interval of already estimated ones.

*Method* 3: Parameter values are estimated using a step-wise approach (similar as *Method 1*), whereby parameter values in upstream levels are fixed when estimating parameters at downstream levels.

Comparison between different model calibration procedures was performed based on the statistical tests and criteria adopted from other studies^59, 60^: (i) goodness-of-fit, using the root mean squared error (RMSE) and mean absolute error (MAE); (ii) a measure to combine model prediction uncertainty and coverage of measurements. This is defined as Length to Coverage of measurements by prediction bands (ILTC). This measure is the ratio of average relative interval length (ARIL)^59^ and coverage of measurements by model uncertainty bands^15^; and (iv) parameter identifiability via parameter uncertainty and parameter correlation analysis^50^.

### Case study of HER and COE drug biomarkers

For the development of the identification method, the transformations of HER and COE drug biomarkers and their respective human metabolites, 6MAM, MORG, MOR and NCOE were used as case study. Specific details regarding the experimental assessment and process model development are presented by Ramin *et al*.^38^. Briefly, the fate of drug biomarkers (partitioning to solids, abiotic and biotic transformation) was assessed in water and untreated wastewater by means of targeted batch experiments (14.3 °C, pH = 8.8) under anaerobic conditions. The chemical transformation kinetics were described by identifying process models (Supplementary Table S1) based on the Activated Sludge Modelling framework for Xenobiotics (ASM-X)^31^. The model includes kinetic parameters, namely abiotic transformation rates, *k*_*abio*_ (d^−1^), and biotic transformation rate constants, *k*_*bio*_ (L gTSS d^−1^), for each selected drug biomarkers. Fate model and model parameters are presented in Supplementary Tables S1 and 2.

## Electronic supplementary material

Supplementary Information
