# Supplementary material for: A systematic model identification method for chemical transformation pathways – the case of heroin biomarkers in wastewater
Source: Sci Rep. 2017 Aug 24;7:9390. doi: 10.1038/s41598-017-09313-y (PMC5571155; doi:10.1038/s41598-017-09313-y)
Supplement: Supplementary file 1 — Supplementary Information [file 41598_2017_9313_MOESM1_ESM.pdf]

Supplementary Information

A systematic model identification method for chemical transformation pathways – the case of heroin biomarkers in wastewater

Pedram Ramin[1,2], Borja Valverde-Pérez[1], Fabio Polesel[1], Luca Locatelli[1], Benedek Gy. Plósz[1,3]

[1]Department of Environmental Engineering, Technical University of Denmark, Bygningstorvet 115, DK 2800 Kgs. Lyngby, Denmark;
[2]Process and Systems Engineering Center (PROSYS), Department of Chemical and Biochemical Engineering, Technical University of Denmark, Building 229, DK-2800 Kgs. Lyngby, Denmark
[3]Department of Chemical Engineering, University of Bath, Claverton Down, Bath BA2 7AY, UK
Correspondence and requests for materials should be addressed to P.R. (pear@kt.dtu.dk) or B.GP. (b.g.plosz@bath.ac.uk)

Number of pages: 11

Number of figures: 5

Number of tables: 4

Supplementary Tables

Table S1. Stoichiometric matrix and process rates for transformation of HER, and it's human metabolites, 6MAM, MORG, MOR also including COE and NCOE, in raw wastewater (suspended biomass) adopted from Ramin et al.[1] Different process rates are associated with different stoichiometry indicated by numbers according to transformation pathway of chemicals, C_{LI}, C_{SL} and C_{SW} are the concentration of drug biomarkers in the liquid phase, attached to suspended solids and attached to reactor wall. Model parameters are defended in Table S2.

Processes	$C_{LI,HER}$	$C_{LI,6MAM}$	$C_{LI,MOR}$	$C_{LI,MORG}$	$C_{LI,COE}$	$C_{LI,NCOE}$	$C_{SL,HER}$	$C_{SL,6MAM}$	$C_{SL,MOR}$	$C_{SL,MORG}$	$C_{SL,COE}$	$C_{SL,NCOE}$	$C_{Sw,HER}$	$C_{Sw,6MAM}$	C_{SwMOR}	$C_{Sw,MORG}$	$C_{Sw,COE}$	$C_{Sw,NCOE}$	Process rates
Abiotic transformation	-1(i)	-1(ii)	-1(iii)	-1(iv)	-1(v)	-1(vi)													$(i)k_{abio,HER}C_{LI,HER}$ $(ii)k_{abio,6MAM}C_{LI,6MAM}$ $(iii)k_{abio,MOR}C_{LI,MOR}$ $(iv)(k_{abio,MORG,1}+k_{abio,MORG,2})C_{LI,MORG}$ $(v)C_{LI,COE,1}(k_{abio,COE,1}+k_{abio,COE,2})$ $(vi)k_{abio,NCOE}C_{LI,NCOE}$
Abiotic formation		1(i)	1(ii)			1(iii)													$(i)k_{abio,HER}C_{LI,HER}(M_{6MAM}/M_{HER})$ $(ii)k_{abio,6MAM}C_{LI,6MAM}(M_{MOR}/M_{6MAM}) + k_{abio,COE,2}C_{LI,COE}(M_{MOR}/M_{6COE})$ $(iii)k_{abio,COE,1}C_{LI,COE}(M_{NCOE}/M_{COE})$
Biotransformation	-1(i)	-1(ii)	-1(iii)	-1(iv)	-1(v)	-1(vi)													$(i)k_{bio,HER}C_{LI,HER}X_{SS}$ $(ii)k_{bio,6MAM}C_{LI,6MAM}X_{SS}$ $(iii)k_{bio,MOR}C_{LI,MOR}X_{SS}$ $(iv)(k_{bio,MORG,1}+k_{bio,MORG,2})C_{LI,MORG}X_{SS}$ $(v)C_{LI,COE,1}(k_{bio,COE,1}+k_{bio,COE,1})\ X_{SS}$ $(vi)k_{abio,NCOE}C_{LI,NCOE}X_{SS}$
Biotic formation		1(i)	1(ii)			1(iii)													$(i)k_{bio,HER}C_{LI,HER}(M_{6MAM}/M_{HER})X_{SS}$ $(ii)k_{bio,6MAM}C_{LI,6MAM}(M_{MOR}/M_{6MAM})X_{SS} + k_{bio,COE,2}C_{LI,COE}(M_{MOR}/M_{COE})X_{SS}$ $(iii)k_{bio,COE,1}C_{LI,COE}(M_{NCOE}/M_{COE})X_{SS}$
De-sorption from wall	1(i)	1(ii)	1(iii)	1(iv)									-1(i)	-1(ii)	-1(iii)	-1(iv)			$(i)K_{des,w,HER}C_{Sw,HER}$ $(ii)K_{des,w,6MAM}C_{Sw,6MAM}$ $(iii)K_{des,w,MOR}C_{Sw,MOR}$ $(iv)K_{des,w,MORG}C_{Sw,MORG}$ $(v)K_{des,w,COE}C_{Sw,COE}$ $(vi)K_{des,w,NCOE}C_{Sw,NCOE}$
Sorption to wall	-1(i)	-1(ii)	-1(iii)	-1(iv)									1(i)	1(ii)	1(iii)	1(iv)			$(i)K_{des,w,HER}K_{d,w,HER}C_{Sw,HER}\alpha_{Sw}$ $(ii)K_{des,w,6MAM}K_{d,w,6MAM}C_{Sw,6MAM}\ \alpha_{Sw}$ $(iii)K_{des,w,MOR}K_{d,w,MOR}C_{Sw,MOR}\alpha_{Sw}$ $(iv)\ K_{des,w,MORG}K_{d,w,MORG}C_{Sw,MORG}\alpha_{Sw}$ $(iii)K_{des,w,COE}K_{d,w,COE}C_{Sw,COE}\alpha_{Sw}$ $(iv)\ K_{des,w,NCOE}K_{d,w,NCOE}C_{Sw,NCOE}\alpha_{Sw}$
De-sorption from suspended solids	1(i)	1(ii)	1(iii)	1(iv)			-1(i)	-1(ii)	-1(iii)	-1(iv)									$(i)K_{des,HER}C_{SL,HER}$ $(ii)K_{des,6MAM}C_{SL,6MAM}$ $(iii)K_{des,MOR}C_{SL,MOR}$ $(iii)K_{des,MORG}C_{SL,MORG}$ $(iv)\ K_{des,COE}C_{SL,COE}$ $(v)\ K_{des,NCOE}C_{SL,NCOE}$
Sorption to suspended solids	-1(i)	-1(ii)	-1(iii)	-1(iv)			1(i)	1(ii)	1(iii)	1(iv)									$(i)k_{des,COC}K_{d,HER}C_{LI,HER}X_{SS}$ $(ii)k_{des,6MAM}K_{d,6MAM}C_{LI,6MAM}X_{SS}$ $(iii)k_{des,MOR}K_{d,MOR}C_{LI,MOR}X_{SS}$ $(iv)k_{des,MORG}K_{d,MORG}C_{LI,MORG}X_{SS}$ $(v)k_{des,COE}K_{d,COE}C_{LI,COE}X_{SS}$ $(vi)k_{des,NCOE}K_{d,NCOE}C_{LI,NCOE}X_{SS}$

Table S2. Fate model parameter for HER and COE drug biomarkers

Symbol	Definition	Unit	HER	MORG	COE	NCOE	6MAM	MOR
$k_{des,w}$	Desorption rate from reactor wall	d^{-1}	100	100	100	100	100	100
K_{dw}	Reactor wall–liquid partition coefficient	L dm^{-2}	0.01	0	0	0.02	0	0.03
k_{des}	Desorption rate from suspended soldis	d^{-1}	100	100	100	100	100	100
K_d	Solid–liquid partition coefficient	L $gTSS^{-1}$	0	0	0	0.01	0.31	0
k_{abio}	Abiotic transformation rate constant	d^{-1}	See Table S3					
k_{bio}	Biotransformation rate constant	L $gTSS^{-1}$ d^{-1}	See Table S3					
M	Biomarker molecular weight	g mol^{-1}	369.41	461.46	299.36	285.34	327.37	285.34
X_{SS}	Concentration of suspended solids	gTSS L^{-1}	0.318					
α_{SW}	wet-surface-to-volume ratio of the reactor	dm^2 L^{-1}	Ramin et al.[1]					

Table S3. Estimated parameters at each level, including, parameter type, pathway, range of parameters and type of distribution at each level for the proposed calibration *Method 1*.

	Parameter type	Model	Pathway	parameter	Min	Max	Distribution
Level 1	Primary	abiotic (A)	HER→6MAM	$k_{abio,HER}$	0	5	uniform
	Combinatorial	abiotic (A)	COE→COE+NCOE	$k_{abio,COE}$	0	1	uniform
Level 2	Primary	abiotic (A)	6MAM→MOR	$k_{abio,6MAM}$	0	1	uniform
		abiotic (A)	COE→NCOE	$k_{abio,COE,1}$	0.0014	0.0843	generalized pareto
		abiotic (A)	COE→MOR	$k_{abio,COE,2}$	0.0064	0.0862	rician
		abiotic (A)	NCOE→T.P.	$k_{abio,NCOE}$	0.0008	0.0843	weibull
	Combinatorial	biotic (B)	HER→6MAM+T.P.	$k_{bio,HER}$	0	3000	uniform
		biotic (B)	MORG→MOR+T.P.	$k_{bio,MORG}$	0	4000	uniform
		biotic (B)	COE→COE+NCOE	$k_{bio,COE}$	0	2	uniform
	Subsidiary	abiotic (A)	HER→6MAM	$k_{abio,HER}$	0.2261	0.5624	loglogistic
		abiotic (A)	COE→COE+NCOE	$k_{abio,COE}$	0.0216	0.0909	tlocationscale
Level 3	Primary	abiotic (A)	MOR→T.P.	$k_{abio,MOR}$	0	1	uniform
		biotic (B)	HER→6MAM	$k_{bio,HER,1}$	0	290.1458	uniform
		biotic (B)	HER→T.P.	$k_{bio,HER,2}$	0	290.1458	uniform
		biotic (B)	6MAM→MOR	$k_{bio,6MAM}$	0	5	uniform
		biotic (B)	COE→ NCOE	$k_{bio,COE,1}$	0	0.2081	uniform
		biotic (B)	COE→MOR	$k_{bio,COE,2}$	0	0.2081	uniform
		biotic (B)	NCOE→T.P.	$k_{bio,NCOE}$	0	1	uniform
	Subsidiary	abiotic (A)	HER→6MAM	$k_{abio,HER}$	0.2261	0.5624	loglogistic
		abiotic (A)	6MAM→MOR	$k_{abio,6MAM}$	0.1023	0.3474	tlocationscale
		abiotic (A)	COE→NCOE	$k_{abio,COE,1}$	0.0014	0.0843	generalized pareto
		abiotic (A)	COE→MOR	$k_{abio,COE,2}$	0.0064	0.0862	rician
		abiotic (A)	NCOE→T.P.	$k_{abio,NCOE}$	0.0008	0.0843	weibull
Level 4	Primary	biotic (B)	MORG→MOR	$k_{bio,MORG,1}$	0	85.4330	uniform
		biotic (B)	MORG→T.P.	$k_{bio,MORG,2}$	0	85.4330	uniform
		biotic (B)	MOR→T.P.	$k_{bio,MOR}$	0	20	uniform
	Subsidiary	abiotic (A)	HER→6MAM	$k_{abio,HER}$	0.2261	0.5624	loglogistic
		abiotic (A)	6MAM→MOR	$k_{abio,6MAM}$	0.1023	0.3474	tlocationscale
		abiotic (A)	MOR→T.P.	$k_{abio,MOR}$	0.0269	0.7047	generalized extreme value
		abiotic (A)	COE→NCOE	$k_{abio,COE,1}$	0.0014	0.0843	generalized pareto
		abiotic (A)	COE→MOR	$k_{abio,COE,2}$	0.0064	0.0862	rician
		abiotic (A)	NCOE→T.P.	$k_{abio,NCOE}$	0.0008	0.0843	weibull
		biotic (B)	HER→6MAM	$k_{bio,HER,1}$	2.9168	33.6060	generalized extreme value
		biotic (B)	HER→T.P.	$k_{bio,HER,2}$	18.0626	54.8897	logistic
		biotic (B)	6MAM→MOR	$k_{bio,6MAM}$	1.1367	3.7540	logistic
		biotic (B)	COE→ NCOE	$k_{bio,COE,1}$	0.0046	0.3925	generalized pareto
		biotic (B)	COE→MOR	$k_{bio,COE,2}$	0.0055	0.4227	generalized pareto
		biotic (B)	NCOE→T.P.	$k_{bio,NCOE}$	0.0177	0.7069	nakagami

Table S4. Estimated parameters using 3 different calibration methods. Estimated values are reported as median with uncertainty reported as 95% credibility interval (lower bound, upper bound). Abbreviations: T.P. = transformation product(s).

	Pathway	Abiotic model parameters, k_{abio} (d^{-1})			Biotic model parameters, k_{bio} (L gTSS d^{-1})		
		method 1	method 2	method 3	method 1	method 2	method 3
Heroin (HER)	HER→ 6MAM	0.351 (0.226,0.562)	2.429 (0.117, 4.889)	0.351 (0.226, 0.562)	15.057 (2.917, 33.606)	99.735 (4.337, 194.178)	16.997 (2.661, 44.769)
	HER→T.P.	0	0	0	38.045 (18.063, 54.890)	135.546 (6.626, 293.389)	34.600 (6.829, 48.937)
6-monoacetylmorphine (6-MAM)	6MAM→ MOR	0.218 (0.102, 0.347)	1.489 (0.104, 4.761)	0.184 (0.110, 0.308)	2.201 (1.137, 3.754)	37.440 (1.322, 97.069)	2.473 (1.219, 6.779)
Morphine-3-β-D-glucuronide (MORG)	MORG→ MOR	0	0	0	11.488 (0.722, 55.372)	115.930 (4.551, 289.899)	15.668 (0.801, 53.730)
	MORG→T.P.	0	0	0	50.672 (11.328, 78.832)	273.698 (18.489, 487.744)	42.533 (4.471, 57.399)
Morphine (MOR)	MOR→T.P.	0.170 (0.027, 0.705)	1.191 (0.149, 3.750)	0.164 (0.035, 0.874)	1.458 (0.077, 14.718)	18.057 (0.729, 48.463)	3.442 (0.205, 18.486)
Codeine (COE)	COE→NCOE	0.044 (0.006, 0.086)	0.406 (0.020, 0.962)	0.036 (0.006, 0.053)	0.114 (0.005, 0.393)	0.835 (0.037, 1.935)	0.110 (0.004, 0.204)
	COE→MOR	0.026 (0.001, 0.084)	0.408 (0.019, 0.959)	0.018 (0.001, 0.048)	0.146 (0.005, 0.423)	0.726 (0.029, 1.934)	0.098 (0.004, 0.204)
Norcodeine (NCOE)	NCOE→T.P.	0.021 (0.001, 0.084)	0.365 (0.022, 0.958)	0.017 (0.001, 0.092)	0.246 (0.018, 0.707)	0.850 (0.043, 1.932)	0.332 (0.056, 0.772)

Supplementary Figures

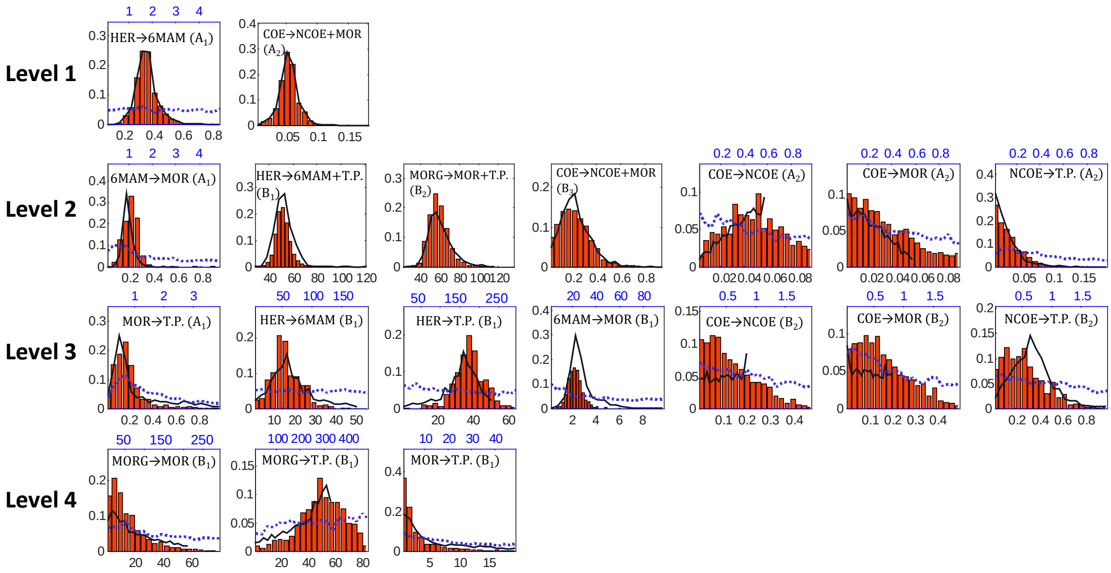

Figure S1. Posterior distribution of estimated parameters (histograms) for proposed methodology-*Method 1* (in red), *Method 2* (dotted blue line, upper X axis) and method 3 (full black line) at 4 different levels. Abbreviations: T.P. = unknown Transformation Product; A= Abiotic model, B= Biotic model.

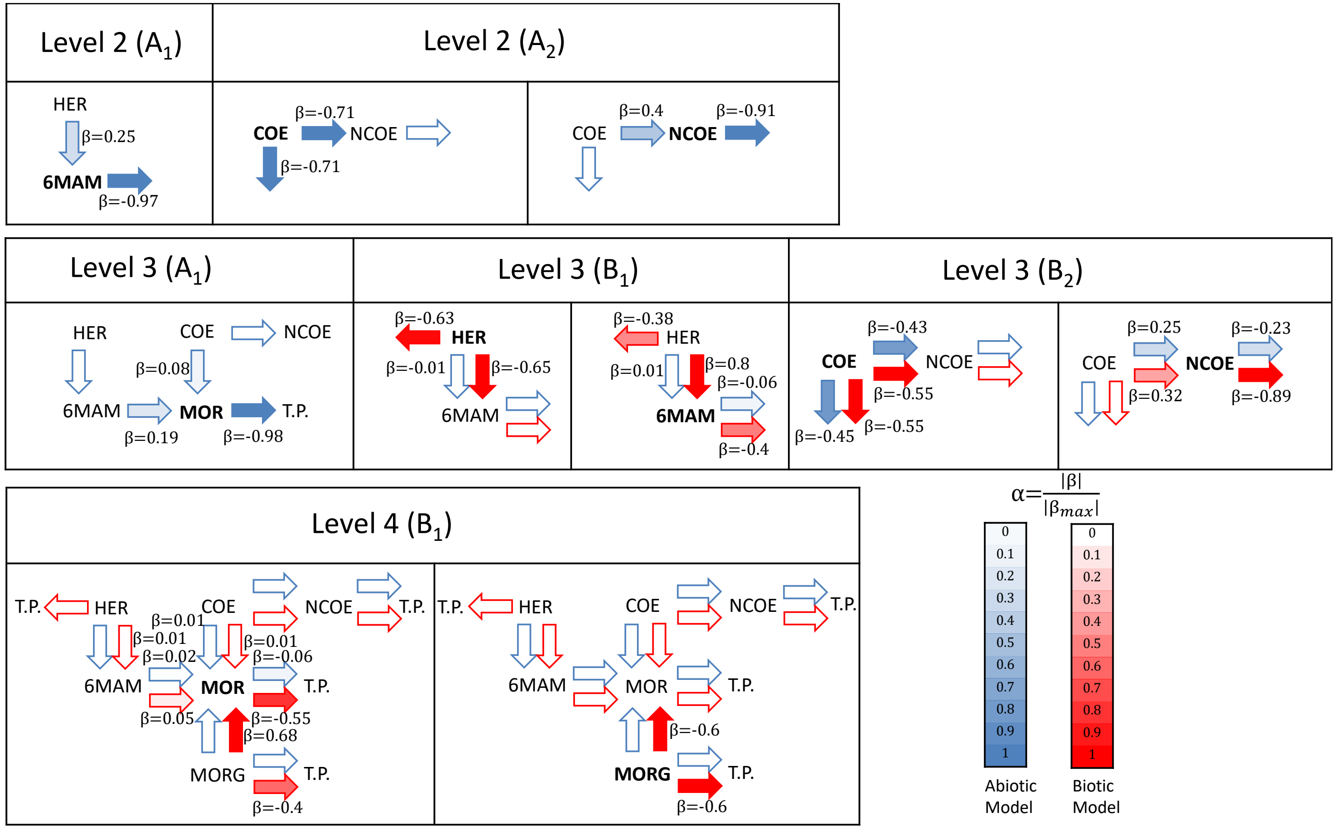

Figure S2. Global sensitivity analysis using standardized regression coefficient (SRC^2) to assess the importance of abiotic and biotic model parameters uncertainty propagation according to *Method 1*. Regression coefficient, β, is used to rank the importance of *subsidiary* parameters on estimation of *primary* parameters at each calibration level. At each level, the sensitivity is explored for one model output, i.e., concentration of a chemical shown in bold. Sensitivity of parameters was used as an indication of error propagation (i.e., higher absolute β value implies more influential propagation indicated using increasing color intensity in each arrow). Direction of blue (abiotic processes) and red (biotic processes) arrows follows the transformation pathways similar to Fig. 1. α is the absolute ratio of β values to a maximum β value (β_{max}) at each level for each chemical, representing color intensities. β values equal to zero are not shown.

		Abiotic Model	
Method 1 Level 2(A_1)		HER→6MAM	6MAM→MOR
Abiotic Model	HER→6MAM	1	
	6MAM→MOR	0.46	1

		Abiotic Model		
Method 1 Level 2(A_2)		COE→NCOE	COE→MOR	NCOE→T.P.
Abiotic Model	COE→NCOE	1		
	COE→MOR	-0.35	1	
	NCOE→T.P.	0.51	-0.09	1

		Abiotic Model	Biotic Model
Method 1 Level 2(B_1)		HER→6MAM	HER→6MAM +T.P.
Abiotic Model	HER→6MAM	1	
Biotic Model	HER→6MAM+T.P.	-0.04	1

		Abiotic Model	Biotic Model
Method 1 Level 2(B_2)		COE→COE+ MOR	COE→COE+ MOR
Abiotic Model	COE→COE+MOR	1	
Biotic Model	COE→COE+MOR	-0.51	1

		Abiotic Model	Biotic Model
Method 1 Level 2(B_3)		HER→6MAM	HER→6MAM +T.P.
Abiotic Model	MORG→MOR	1	
Biotic Model	MORG→MOR+T.P.	0	1

		Abiotic Model						
Method 1 Level 3(A_1)		HER→6MAM	6MAM→MOR	MOR→T.P.	MORG→MOR	COE→NCOE	COE→MOR	NCOE→T.P.
Abiotic Model	HER→6MAM	1						
	6MAM→MOR	0.22	1					
	MOR→T.P.	0.11	0.08	1				
	MORG→MOR				1			
	COE→NCOE	0.03	-0.01	0.02		1		
	COE→MOR	0.01	0.01	0.22		-0.06	1	
	NCOE→T.P.	0.02	0.03	0.05		0.24	-0.03	1

		Abiotic Model		Biotic Model		
Method 1 Level 3(B_1)		HER→6MAM	6MAM→MOR	HER→6MAM	HER→T.P.	6MAM→MOR
Abiotic Model	HER→6MAM	1				
	6MAM→MOR	0.16	1			
Biotic Model	HER→6MAM	-0.09	0.04	1		
	HER→T.P.	0.05	-0.02	-0.67	1	
	6MAM→MOR	-0.10	-0.28	0.66	-0.63	1

		Abiotic Model			Biotic Model		
Method 1 Level 3(B_2)		COE→NCOE	COE→MOR	NCOE→T.P.	COE→NCOE	COE→MOR	NCOE→T.P.
Abiotic Model	COE→NCOE	1					
	COE→MOR	-0.02	1				
	NCOE→T.P.	0.07	-0.03	1			
Biotic Model	COE→NCOE	-0.03	-0.08	-0.01	1		
	COE→MOR	-0.06	-0.08	-0.03	-0.45	1	
	NCOE→T.P.	0.33	-0.07	-0.32	0.53	-0.25	1

		Abiotic Model							Biotic Model								
Method 1 Level 4(B_1)		HER→6MAM	6MAM→MOR	MOR→T.P.	MORG→MOR	COE→NCOE	COE→MOR	NCOE→T.P.	HER→6MAM	HER→T.P.	6MAM→MOR	MOR→T.P.	MORG→MOR	MORG→T.P.	COE→NCOE	COE→MOR	NCOE→T.P.
Abiotic Model	HER→6MAM	1															
	6MAM→MOR	0.07	1														
	MOR→T.P.	0.04	0.08	1													
	MORG→MOR				1												
	COE→NCOE	0.07	0.01	0.00		1											
	COE→MOR	-0.03	0.03	0.01		0.02	1										
	NCOE→T.P.	0.06	0.05	0.05		0.01	-0.03	1									
Biotic Model	HER→6MAM	0.00	-0.04	-0.01		0.05	-0.06	-0.05	1								
	HER→T.P.	0.00	-0.06	0.00		0.03	-0.03	0.02	-0.01	1							
	6MAM→MOR	-0.02	-0.10	0.03		-0.10	-0.02	-0.01	0.06	0.00	1						
	MOR→T.P.	0.00	0.04	0.02		0.03	0.01	0.02	0.01	-0.06	-0.03	1					
	MORG→MOR	0.01	0.03	0.12		0.05	0.01	0.03	-0.03	0.01	-0.13	0.60	1				
	MORG→T.P.	0.05	-0.04	-0.12		-0.01	-0.03	0.03	0.03	0.06	0.12	-0.52	-0.69	1			
	COE→NCOE	0.02	0.03	0.04		-0.07	0.01	0.09	0.02	-0.02	-0.05	0.15	0.14	-0.11	1		
	COE→MOR	-0.02	0.02	0.03		-0.01	-0.02	0.03	0.04	0.00	0.01	0.16	0.10	-0.10	0.01	1	
	NCOE→T.P.	0.08	-0.04	0.05		0.18	0.03	-0.20	0.06	0.00	-0.06	0.06	0.09	-0.03	0.29	0.01	1

Figure S3. Co-linearity matrix between model parameters at each level for *Method 1*. Parameters in the shadings are the ones calibrated at each level.

Method 2 Abiotic Model		Abiotic Model						
		HER→6MAM	6MAM→MOR	MOR→T.P.	MORG→MOR	COE→NCOE	COE→MOR	NCOE→T.P.
Abiotic Model	HER→6MAM	1						
	6MAM→MOR	0.00	1					
	MOR→T.P.	0.04	0.22	1				
	MORG→MOR				1			
	COE→NCOE	0.01	0.05	0.07		1		
	COE→MOR	-0.02	0.10	0.17		0.11	1	
	NCOE→T.P.	0.01	0.12	0.15		0.25	0.04	1

Method 2 Biotic Model		Abiotic Model							Biotic Model								
		HER→6MAM	6MAM→MOR	MOR→T.P.	MORG→MOR	COE→NCOE	COE→MOR	NCOE→T.P.	HER→6MAM	HER→T.P.	6MAM→MOR	MOR→T.P.	MORG→MOR	MORG→T.P.	COE→NCOE	COE→MOR	NCOE→T.P.
Abiotic Model	HER→6MAM	1															
	6MAM→MOR	-0.02	1														
	MOR→T.P.	0.01	0.04	1													
	MORG→MOR				1												
	COE→NCOE	0.04	0.05	0.01		1											
	COE→MOR	-0.02	0.01	0.00		0.10	1										
	NCOE→T.P.	0.03	0.01	-0.03		0.22	0.03	1									
Biotic Model	HER→6MAM	0.04	-0.01	-0.01		0.00	0.01	-0.01	1								
	HER→T.P.	0.01	-0.03	-0.02		-0.01	0.05	-0.03	0.00	1							
	6MAM→MOR	-0.01	0.06	0.03		0.03	0.02	0.02	-0.03	0.00	1						
	MOR→T.P.	0.04	0.04	0.05		0.04	0.07	0.00	0.03	0.02	0.19	1					
	MORG→MOR	0.00	0.01	0.03		0.02	0.05	0.02	0.00	0.02	-0.02	0.14	1				
	MORG→T.P.	-0.04	0.01	-0.06		0.01	-0.03	0.00	-0.03	-0.04	0.03	-0.16	0.11	1			
	COE→NCOE	0.03	0.00	0.00		-0.06	0.05	0.18	-0.03	-0.01	0.08	0.10	0.04	-0.10	1		
	COE→MOR	0.00	0.03	-0.01		0.07	0.04	-0.05	-0.01	0.04	0.07	0.09	0.00	-0.02	-0.01	1	
	NCOE→T.P.	0.05	0.04	0.07		0.18	0.01	-0.19	0.00	0.03	0.04	0.05	0.05	-0.06	0.05	0.06	1

Figure S4. Co-linearity matrix between for abiotic and biotic model parameters for *Method 2*. Parameters in the shadings are the ones calibrated at each level.

Method 3 Level 2(A_1)		Abiotic Model: HER→6MAM	Abiotic Model: 6MAM→MOR
Abiotic Model	HER→6MAM	1	
Abiotic Model	6MAM→MOR	0	1

Method 3 Level 2(A_2)		Abiotic Model: COE→NCOE	Abiotic Model: COE→MOR	Abiotic Model: NCOE→T.P.
Abiotic Model	COE→NCOE	1		
Abiotic Model	COE→MOR	-1	1	
Abiotic Model	NCOE→T.P.	0.19	-0.19	1

Method 3 Level 2(B_1)		Abiotic Model: HER→6MAM	Biotic Model: HER→6MAM +T.P.
Abiotic Model	HER→6MAM	1	
Biotic Model	HER→6MAM+T.P.		1

Method 3 Level 2(B_2)		Abiotic Model: COE→COE+ MOR	Biotic Model: COE→COE+ MOR
Abiotic Model	COE→COE+MOR	1	
Biotic Model	COE→COE+MOR		1

Method 3 Level 2(B_3)		Abiotic Model: HER→6MAM	Biotic Model: HER→6MAM +T.P.
Abiotic Model	MORG→MOR	1	
Biotic Model	MORG→MOR+T.P.		1

Method 1 Level 3(A_1)		Abiotic Model: HER→6MAM	6MAM→MOR	MOR→T.P.	MORG→MOR	COE→NCOE	COE→MOR	NCOE→T.P.
Abiotic Model	HER→6MAM	1						
Abiotic Model	6MAM→MOR		1					
Abiotic Model	MOR→T.P.			1				
Abiotic Model	MORG→MOR				1			
Abiotic Model	COE→NCOE					1		
Abiotic Model	COE→MOR						1	
Abiotic Model	NCOE→T.P.							1

Method 3 Level 3(B_1)		Abiotic Model: HER→6MAM	Abiotic Model: 6MAM→MOR	Biotic Model: HER→6MAM	Biotic Model: HER→T.P.	Biotic Model: 6MAM→MOR
Abiotic Model	HER→6MAM	1				
Abiotic Model	6MAM→MOR		1			
Biotic Model	HER→6MAM			1		
Biotic Model	HER→T.P.			-1	1	
Biotic Model	6MAM→MOR			-0.7	-0.7	1

Method 3 Level 3(B_2)		Abiotic Model: COE→NCOE	Abiotic Model: COE→MOR	Abiotic Model: NCOE→T.P.	Biotic Model: COE→NCOE	Biotic Model: COE→MOR	Biotic Model: NCOE→T.P.
Abiotic Model	COE→NCOE	1					
Abiotic Model	COE→MOR		1				
Abiotic Model	NCOE→T.P.			1			
Biotic Model	COE→NCOE				1		
Biotic Model	COE→MOR				-1	1	
Biotic Model	NCOE→T.P.				0.40	-0.40	1

Method 3 Level 4(B_1)		Abiotic Model: HER→6MAM	6MAM→MOR	MOR→T.P.	MORG→MOR	COE→NCOE	COE→MOR	NCOE→T.P.	Biotic Model: HER→6MAM	HER→T.P.	6MAM→MOR	MOR→T.P.	MORG→MOR	MORG→T.P.	COE→NCOE	COE→MOR	NCOE→T.P.
Abiotic Model	HER→6MAM	1															
Abiotic Model	6MAM→MOR		1														
Abiotic Model	MOR→T.P.			1													
Abiotic Model	MORG→MOR				1												
Abiotic Model	COE→NCOE					1											
Abiotic Model	COE→MOR						1										
Abiotic Model	NCOE→T.P.							1									
Biotic Model	HER→6MAM								1								
Biotic Model	HER→T.P.									1							
Biotic Model	6MAM→MOR										1						
Biotic Model	MOR→T.P.											1					
Biotic Model	MORG→MOR											0.56	1				
Biotic Model	MORG→T.P.											-0.56	-1	1			
Biotic Model	COE→NCOE														1		
Biotic Model	COE→MOR															1	
Biotic Model	NCOE→T.P.																1

Figure S5. Co-linearity matrix between for abiotic and biotic model parameters for *Method*. Parameters in the shadings are the ones calibrated at each level.

References

1. Ramin, P., Brock, A. L., Polesel, F., Causanilles, A., Emke, E., de Voogt, P. & Plósz, B. G. Transformation and sorption of illicit drug biomarkers in sewer systems: understanding the role of suspended solids in raw wastewater. *Environ. Sci. Technol.* 2016, ***50*** (24), 13397–13408.

2. Sin, G., Gernaey, K. V., Neumann, M. B., van Loosdrecht, M. C. M. & Gujer, W. Global sensitivity analysis in wastewater treatment plant model applications: Prioritizing sources of uncertainty. *Water Res.* **45,** 639–651 (2011).
